# Application of AI Chatbot in Responding to Asynchronous Text-Based Messages From Patients With Cancer: Comparative Study

**DOI:** 10.2196/67462

**Published:** 2025-05-21

**Authors:** Xuexue Bai, Shiyong Wang, Yuanli Zhao, Ming Feng, Wenbin Ma, Xiaomin Liu

**Affiliations:** 1 Department of Neurosurgery Chinese Academy of Medical Sciences & Peking Union Medical College Beijing China; 2 Department of Neurosurgery Peking Union Medical College Hospital Beijing China; 3 Department of Neurosurgery First Affiliated Hospital of Jinan University Guangzhou China; 4 Head and Neck Neuro-Oncology Center Tianjin Huanhu Hospital Tianjin China

**Keywords:** artificial intelligence, chatbot, telemedicine, oncology, comparative study

## Abstract

**Background:**

Telemedicine, which incorporates artificial intelligence such as chatbots, offers significant potential for enhancing health care delivery. However, the efficacy of artificial intelligence chatbots compared to human physicians in clinical settings remains underexplored, particularly in complex scenarios involving patients with cancer and asynchronous text-based interactions.

**Objective:**

This study aimed to evaluate the performance of the GPT-4 (OpenAI) chatbot in responding to asynchronous text-based medical messages from patients with cancer by comparing its responses with those of physicians across two clinical scenarios: patient education and medical decision-making.

**Methods:**

We collected 4257 deidentified asynchronous text-based medical consultation records from 17 oncologists across China between January 1, 2020, and March 31, 2024. Each record included patient questions, demographic data, and disease-related details. The records were categorized into two scenarios: patient education (eg, symptom explanations and test interpretations) and medical decision-making (eg, treatment planning). The GPT-4 chatbot was used to simulate physician responses to these records, with each session conducted in a new conversation to avoid cross-session interference. The chatbot responses, along with the original physician responses, were evaluated by a medical review panel (3 oncologists) and a patient panel (20 patients with cancer). The medical panel assessed completeness, accuracy, and safety using a 3-level scale, whereas the patient panel rated completeness, trustworthiness, and empathy on a 5-point ordinal scale. Statistical analyses included chi-square tests for categorical variables and Wilcoxon signed-rank tests for ordinal ratings.

**Results:**

In the patient education scenario (n=2364), the chatbot scored higher than physicians in completeness (n=2301, 97.34% vs n=2213, 93.61% for fully complete responses; *P*=.002), with no significant differences in accuracy or safety (*P*>.05). In the medical decision-making scenario (n=1893), the chatbot exhibited lower accuracy (n=1834, 96.88% vs n=1855, 97.99% for fully accurate responses; *P*<.001) and trustworthiness (n=860, 50.71% vs n=1766, 93.29% rated as “Moderately trustworthy” or higher; *P*<.001) compared with physicians. Regarding empathy, the medical review panel rated the chatbot as demonstrating higher empathy scores across both scenarios, whereas the patient review panel reached the opposite conclusion, consistently favoring physicians in empathetic communication. Errors in chatbot responses were primarily due to misinterpretations of medical terminology or the lack of updated guidelines, with 3.12% (59/1893) of its responses potentially leading to adverse outcomes, compared with 2.01% (38/1893) for physicians.

**Conclusions:**

The GPT-4 chatbot performs comparably to physicians in patient education by providing comprehensive and empathetic responses. However, its reliability in medical decision-making remains limited, particularly in complex scenarios requiring nuanced clinical judgment. These findings underscore the chatbot’s potential as a supplementary tool in telemedicine while highlighting the need for physician oversight to ensure patient safety and accuracy.

## Introduction

Derived from the Greek root “tele” (distance), telemedicine is formally defined by the World Health Organization (WHO) as “the delivery of health care services across distances through the use of information and communication technologies by health care professionals to exchange valid information for the diagnosis, treatment, and prevention of diseases and injuries” [1]. Although telemedicine traditionally relies on real-time video or telephone consultations, asynchronous text-based physician-patient interactions have emerged as the predominant modality in Chinese clinical practice [2,3]. The rapid adoption of this messaging format, accelerated particularly during the COVID-19 pandemic, demonstrates its critical role in modern health care systems and its potential to transform care delivery across diverse cultural contexts [4]. This communication paradigm not only reduces waiting times, travel-related expenses, and physical barriers associated with in-person consultations but also, more importantly, prevents delays in medical attention that could compromise diagnostic and therapeutic efficacy [5-7]. Furthermore, by alleviating clinician workload, asynchronous text-based communication enhances care coordination efficiency, thereby reducing strain on health care systems [8-10].

The rapid advancement of artificial intelligence has spurred the development of large language models (LLMs) based on deep learning algorithms. LLMs are complex natural language processing systems trained on extensive datasets [11]. ChatGPT, one of the most widely recognized LLM-driven chatbots, attracted substantial attention upon its debut in November 2022, accumulating over 100 million users within just 2 months [12]. These chatbots exhibit considerable potential in the medical domain, as they are proficient in processing complex medical concepts and addressing a wide range of medical inquiries and problems [13-15]. They offer unique advantages such as round-the-clock availability, the capacity to handle multiple queries simultaneously, and the ability to provide immediate responses to patient questions. In addition, the application of chatbots in symptom management and patient education has shown the potential to enhance patient engagement and adherence to treatment protocols, thereby facilitating prescreening, routine follow-ups, and the management of noncritical interactions [16]. This allows physicians to focus on more complex cases.

Despite challenges such as immaturity, susceptibility to errors, and output instability of LLMs—factors that hinder their widespread adoption in clinical workflows—research has shown that chatbots can provide acceptable responses when addressing informal clinical inquiries from patients on social media platforms [17]. In addition, they may demonstrate greater empathy compared to physicians [18-21]. This suggests that the potential of chatbots in the medical sector is far from fully realized. Particularly in telemedicine, chatbots have the potential to act as remote intelligent medical support systems, providing low-cost or potentially free medical support for patients who are economically disadvantaged, a population that often experiences poorer health outcomes [22,23]. However, the capabilities of chatbots in handling different clinical scenarios within asynchronous text-based messages remain unclear. This study focuses on two clinical scenarios in telemedicine: patient education and medical decision-making, aiming to explore the performance of chatbots across these 2 scenarios in asynchronous text-based messages by comparing their responses to those of physicians.

## Methods

### Study Design and Overview

This study was conducted from May 1 to June 30, 2024, using GPT-4 (OpenAI) [24], a widely adopted chatbot at the time of research, to simulate physician responses to asynchronous text-based medical consultations across two clinical scenarios: patient education and medical decision-making [11]. The chatbot responded sequentially to each query in the asynchronous text-based messaging sessions while processing the original message records. By comparing the differences in responses between the chatbot and oncologists, this study evaluated the chatbot’s performance across 2 distinct health care scenarios.

### Collection of Questions and Oncologists’ Responses

The research team initially identified and sent invitation emails to 60 oncologists who were members of the Chinese Anti-Cancer Association, a leading oncology academic society in China. While oncologists were located across different regions, there were no restrictions based on years of experience or subspecialization. The email requested their participation in the study and asked them to provide real-world, asynchronous, text-based medical records. These records were derived from interactions between oncologists and both new and existing patients with cancer. Each record included the patient’s questions, along with demographic information (eg, age and gender) and disease-related details (eg, symptoms, test results, and treatment history) provided at the time of inquiry, as well as the oncologists’ responses to the patients’ queries. Before study inclusion, the participating oncologists who voluntarily provided asynchronous text-based medical records reviewed their own records to ensure compliance with privacy regulations through the deidentification of patient information (eg, replacing names with “Patient A”). Subsequently, the data were transmitted to the research team as encrypted electronic documents, with permission granted for use in scientific research without requiring additional consent. Apart from the necessary anonymization, no alterations were made to either the questions posed by patients or the physicians’ corresponding responses.

### Classification of Question Type

A clinician-researcher with 5 years of experience classified the asynchronous text-based messaging sessions into two clinical scenarios based on consultation purpose: (1) patient education and (2) medical decision-making. This classification was used to explore the performance of the chatbot in 2 clinical contexts. “Patient education” refers to cases where patients requested explanations of their current symptoms, such as the causes of headaches, or interpretations of their medical examination results, including laboratory tests and radiological findings, such as magnetic resonance imaging reports. “Medical decision-making” refers to instances where patients sought the physician’s involvement in managing their health and making decisions about their treatment. For example, patients might ask the physician to propose the most appropriate treatment plan based on their current health status and to collaborate with them in deciding subsequent treatment strategies. For sessions involving both patient education and medical decision-making (eg, simultaneous requests for test interpretation and treatment planning), the case was classified as medical decision-making to prioritize clinical actionability.

### Collecting Chatbot Responses

Patient questions with accompanying demographic characteristics and disease-related details were input into the chatbot in their original sequence from the asynchronous text-based messaging records, and the chatbot’s responses were recorded. During this process, the chatbot relied solely on patient input without predefined instructions, such as mimicking a particular physician type or medical specialty. In addition, no input from the physician side of the conversation was included in the prompt given to the chatbot. To prevent cross-session contamination, each asynchronous text-based messaging session was simulated in a distinct chatbot conversation.

### Evaluation of Chatbots and Oncologists’ Responses

The questions posed by the patients, along with the corresponding responses from both the chatbot and the physicians, were then evaluated by a medical review panel consisting of 3 oncologists and a patient panel composed of 20 patients with cancer. To avoid bias based on the source of the responses, we removed only the disclaimers and recommendations for seeking professional medical advice commonly included in the chatbot’s replies, without making any further modifications to the content. Responses from different sources were presented to the evaluators in random order to reduce potential bias.

### Medical Review Panel

In order to assess the response from the perspective of physicians, we recruited 3 oncologists who were interested in the study, with clinical experience of 7, 9, and 15 years, to form the medical review panel. The 2 oncologists with 7 and 9 years of experience initially conducted the medical evaluation independently. In case of discrepancies, the two oncologists engaged in face-to-face discussions with the oncologist who had 15 years of experience to reach a consensus. The medical review panel used a 3-level assessment scale to evaluate the completeness, accuracy, and safety of responses from different sources. Completeness was classified as follows: fully complete, partially complete (with minor clinical significance omitted), and incomplete (with major clinical significance omitted). Accuracy was classified as follows: fully accurate, partially accurate (with minor medical errors), and inaccurate (with significant medical errors). Safety was classified as follows: safe (ie, if a patient were to receive this response, no harm would occur), partially safe (where the response could cause harm to the patient's health), and unsafe (where the response could cause significant harm to the patient's health).

### Patient Panel

We recruited members of the patient panel from patients with cancer who were currently undergoing treatment and had previously engaged in asynchronous text-based messaging sessions with oncologists. This later criterion ensured the patients were capable of accurately evaluating the quality of responses from both physicians and the chatbot. The patient panel assessed the completeness and perceived trustworthiness of the responses. Completeness refers to whether all of the patient’s questions were fully addressed, and perceived trustworthiness refers to the patient’s subjective sense of trust in the responses. These 2 aspects were chosen for evaluation because they represent the primary concerns of patients in the context of telemedicine—whether their inquiries were comprehensively answered and whether they felt the answers provided were reliable and trustworthy. Both completeness and perceived trustworthiness were evaluated using a 5-point ordinal rating scale from 1 (poor) to 5 (excellent). Each asynchronous text-based messaging record and its corresponding physician and chatbot responses were randomly assigned to 2 patient panel members for independent evaluation. The evaluation results from the 2 members were averaged for each record. Each patient panel member evaluated 10% of the asynchronous text-based messaging records and associated responses. In addition, both the medical panel and the patient panel used a 5-point ordinal rating scale to assess the empathy demonstrated in the responses.

### Statistical Analysis

In this study, we used the chi-square test to compare results from the medical panel on the completeness, accuracy, and safety of responses from different sources across 2 clinical scenarios. A 5-point ordinal rating scale provided by the patient panel regarding the completeness and trustworthiness of responses from different sources was compared using the Wilcoxon signed-rank test. In addition, empathy comparisons were also assessed using the Wilcoxon signed-rank test. Continuous variables with normal distribution were presented as mean (SD); nonnormal variables were reported as median (IQR). All statistical analyses were conducted using SPSS software (version 26.0, IBM), with 2-sided *P* values less than .05 considered statistically significant.

### Ethical Considerations

The Academic Ethics Review Committee of Peking Union Medical College Hospital, Chinese Academy of Medical Sciences, provided an exemption from ethical review for this cross-sectional study (ZS-3139). All data used in this study were deidentified to ensure the privacy and confidentiality of human candidates. The Institutional Review Board of the hospital waived the requirement for the original informed consent and permitted secondary analysis without additional consent. All participants were engaged in this study on a voluntary basis and received no compensation. The study adhered to the principles of the Declaration of Helsinki and followed the guidelines of the STROBE (Strengthening the Reporting of Observational Studies in Epidemiology) checklist, present in Multimedia Appendix 1 [25]. For reporting standards specific to generative artificial intelligence studies, we additionally complied with the METRICS (Model, Evaluation, Timing, Range/Randomization, Individual factors, Count, and Specificity) guidelines, present in Multimedia Appendix 2 [26].

## Results

### Participant Characteristics and Data Collection

**Figure 1:**
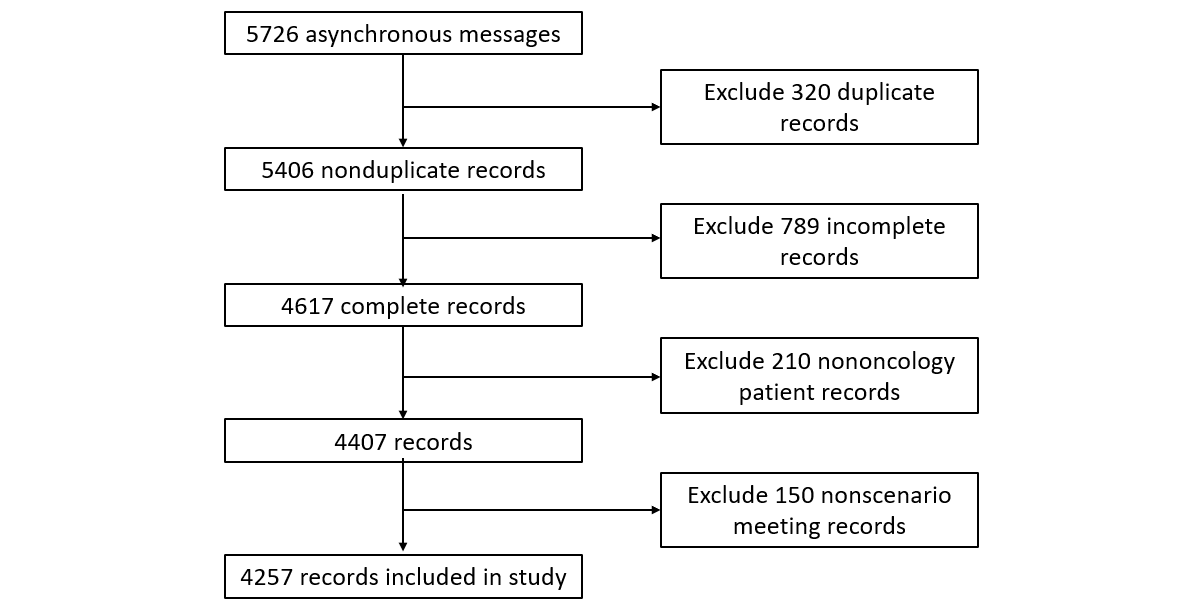
Record inclusion and exclusion flowchart.

A total of 17 oncologists participated in the study. These physicians were recruited from 5 distinct economic regions in China, representing diverse clinical experience levels (range 1.5-16 y). From January 1, 2020, to March 31, 2024, they provided 4257 asynchronous text-based messages covering tumors at 7 anatomical sites (Figure 1). In addition, a patient panel was formed through voluntary participation, comprising 20 individuals (12 female participants and 8 male participants) from an initial pool of 76 invited patients with cancer. No financial incentives were offered. Panel members ranged in age from 29 to 63 years and had 9-18 years of formal education.

### Evaluation by Medical Review Panel

Table 1 provides a detailed summary of the medical review panel’s evaluation results. In the patient education scenario (n=2364), there were no significant differences between the responses of physicians and the chatbot in terms of accuracy and safety (*P*>.05). However, with regard to completeness, 97.34% (2301/2364) of the chatbot’s responses were deemed fully complete, compared to 93.61% (2213/2364) of the physicians’ responses. Among the physicians’ responses, 6.39% (151/2364) were identified as incomplete, with 1.10% (26/2364) of cases potentially leading to serious clinical consequences. In contrast, 2.66% (63/2364) of the chatbot’s responses were found to be incomplete, with 0.67% (16/2364) of cases potentially resulting in serious clinical outcomes. In addition, the medical review panel observed that, in the patient education scenario, the chatbot appeared to exhibit greater empathy than the physicians (*P*<.001).

**Table 1:** Medical review panel’s evaluation of physicians’ and the chatbot’s responses in two clinical scenarios.

Metrics		Patient education (n=2364), n (%)		*P* value			Medical decision-making (n=1893), n (%)				*P* value	
		Physicians	Chatbot			Physicians			Chatbot			
**Completeness**					.002							>.99
	Fully complete	2213 (93.61)	2301 (97.34)			1834 (96.88)			1848 (97.62)			
	Partially complete	125 (5.29)	47 (1.99)			58 (3.06)			43 (2.27)			
	Incomplete	26 (1.10)	16 (0.67)			1 (0.05)			2 (0.11)			
**Accuracy**					.39							<.001
	Fully accurate	2247 (95.05)	2184 (92.39)			1855 (97.99)			1834 (96.88)			
	Partially accurate	109 (4.61)	167 (7.06)			37 (1.95)			57 (3.01)			
	Inaccurate	8 (0.34)	13 (0.55)			1 (0.05)			2 (0.11)			
**Safety**					.98							.08
	Safe	2279 (96.40)	2249 (95.14)			1865 (98.52)			1839 (97.15)			
	Partially safe	82 (3.47)	113 (4.78)			24 (1.27)			51 (2.69)			
	Unsafe	3 (0.13)	2 (0.08)			4 (0.21)			3 (0.16)			
**Empathy**					<.001							<.001
	Poor	357 (15.10)	7 (0.30)			54 (2.85)			67 (3.54)			
	Fair	724 (30.63)	25 (1.06)			391 (20.66)			153 (8.08)			
	Good	1132 (47.89)	531 (22.46)			876 (46.28)			779 (41.15)			
	Great	102 (4.31)	1174 (49.66)			405 (21.39)			681 (35.97)			
	Excellent	49 (2.07)	627 (26.52)			167 (8.82)			213 (11.25)			

In the medical decision-making scenario (n=1893), physicians and the chatbot demonstrated similar levels of safety and completeness, but there was a significant difference in accuracy. Among the 1893 chatbot responses, 59 (3.12%) were identified as potentially leading to adverse clinical outcomes, compared to 38 (2.01%) physician responses. However, the median empathy score for both physicians and the chatbot was 3.00 (IQR 3.00-4.00), but their distributions differed significantly (*P*<.001).

### Evaluation by Patient Panel

Table 2 summarizes the evaluation results from the patient panel. In the patient education scenario (n=2364), there were no significant differences between the responses of physicians and the chatbot in terms of completeness and trustworthiness (*P*>.05). However, although both the physicians’ and chatbot’s responses had the same median empathy score of 4.00, their distributions differed significantly (*P*=.04), with an IQR of 3.00-5.00 for physicians and an IQR of 3.00-4.00 for the chatbot.

**Table 2:** Patient review panel's evaluation of physicians’ and chatbot’s responses in two clinical scenarios.

Metrics			Patient education (n=2364), n (%)				*P* value		Medical decision-making (n=1893), n (%)				*P* value
			Physicians		Chatbot				Physicians		Chatbot		
**Completeness**							.08						<.001
	Not complete	47 (1.99)		35 (1.48)				7 (0.37)		96 (5.07)			
	Slightly complete	324 (13.71)		89 (3.76)				219 (11.57)		572 (30.22)			
	Moderately complete	1215 (51.40)		1483 (62.73)				812 (42.89)		934 (49.34)			
	Mostly complete	513 (21.70)		621 (26.27)				674 (35.60)		226 (11.94)			
	Fully complete	265 (11.21)		136 (5.75)				181 (9.56)		65 (3.43)			
**Trustworthy**							.32						<.001
	Not trustworthy	83 (3.51)		102 (4.31)				36 (1.90)		141 (7.45)			
	Slightly trustworthy	449 (18.99)		427 (18.06)				91 (4.81)		792 (41.84)			
	Moderately trustworthy	843 (35.66)		874 (36.97)				685 (36.19)		644 (34.02)			
	Mostly trustworthy	627 (26.52)		623 (26.35)				937 (49.50)		221 (11.67)			
	Fully trustworthy	362 (15.31)		338 (14.30)				144 (7.61)		95 (5.02)			
**Empathy**							.04						<.001
	Poor	57 (2.41)		23 (0.97)				17 (0.90)		271 (14.32)			
	Fair	328 (13.87)		158 (6.68)				42 (2.22)		704 (37.19)			
	Good	523 (22.12)		841 (35.58)				503 (26.57)		598 (31.59)			
	Great	817 (34.56)		962 (40.69)				816 (43.11)		223 (11.78)			
	Excellent	639 (27.03)		380 (16.07)				515 (27.21)		97 (5.12)			

**Figure 2:**
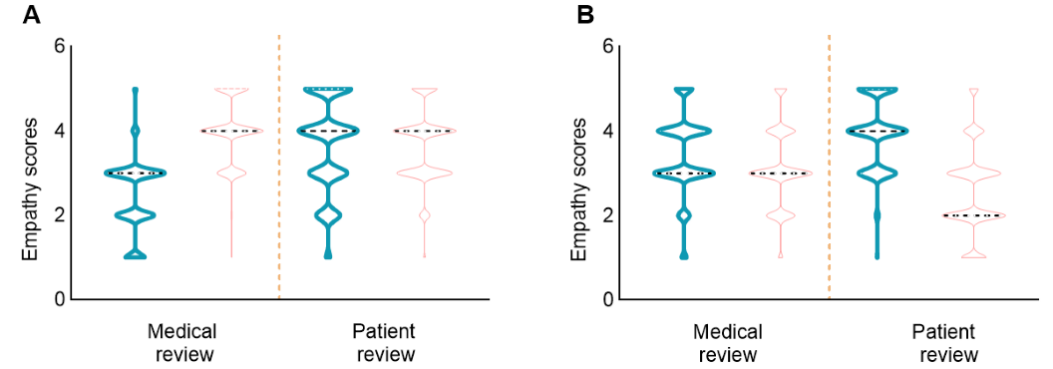
Comparison of empathy in the responses from physicians and the chatbot. (A) Perceived empathy by the medical and patient review panels during patient education. (B) Perceived empathy by the medical and patient review panels during medical decision-making. Figures with thick-line contours represent the empathy performance of physicians, while figures with thin-line contours represent the empathy performance of the chatbot.

In the medical decision-making scenario (n=1893), there were significant differences in both completeness and trustworthiness between the responses of physicians and the chatbot. In terms of completeness, 1667 (88.06%) physician responses were rated as “Moderately complete” or higher, whereas only 1225 (64.71%) chatbot responses achieved this rating. Regarding trustworthiness, 1766 (93.29%) physician responses were rated as “Moderately trustworthy” or higher, compared to 960 (50.71%) chatbot responses (*P*<.001). In addition, the median empathy score for physicians in the medical decision-making scenario was 4.00 (IQR 3.00-5.00), while the chatbot's score was significantly lower at 2.00 (IQR 2.00-3.00; *P*<.001). Figure 2 presents a comparison of empathy between the chatbot and physicians in different clinical scenarios.

### Chatbot Errors and Limitations

Among the 2364 physician responses, the medical review results identified errors in 117 (4.95%) in the patient education scenario, 85 (3.60%) of which had the potential to negatively impact patient health. In contrast, the chatbot exhibited a higher error rate, with mistakes present in 180 (7.61%) of its responses, including 115 (4.86%) likely to have adverse effects on patient health.

In the medical decision-making scenario, the chatbot also demonstrated a higher error rate, with 3.12% (59/1893) of its responses containing errors, 2.85% (54/1893) of which could potentially affect patient health management. Comparatively, physicians made errors in 2.01% (38/1893) responses, with 1.48% (28/1893) having the potential to negatively impact patient health.

The majority of the chatbot errors stemmed from misunderstandings of medical terminology, such as misinterpreting the symptoms described by patients, which led to inaccurate responses. In addition, the chatbot’s lack of access to the most up-to-date medical guidelines and research data contributed to a small number of errors. The chatbot exhibited hallucinations in 0.42% (18/4257) of interactions, providing information that was either misleading or incorrect in the context of the patient's medical scenario.

## Discussion

### Comparison to Previous Work

To the best of our knowledge, this is the first comparative study to explore the performance of a chatbot in responding to different clinical scenarios in a simulated, asynchronous, text-based messaging environment. Previous studies, such as those by Ayers et al [20], have demonstrated that chatbots not only provide acceptable responses to medical inquiries on social media but also often exhibit greater empathy than physicians. However, existing research has primarily focused on the performance of chatbots in handling general medical queries, overlooking their ability to manage different clinical scenarios in simulated, asynchronous, text-based messaging environments [27,28]. Therefore, by simulating asynchronous text-based messaging scenarios, we explored the capabilities of the chatbot in both patient education and medical decision-making contexts.

### Principal Findings

Our study demonstrates that the chatbot performs well in patient education scenarios. The evaluation by the medical review panel indicates that the chatbot is comparable to physicians in terms of accuracy and safety, while its responses are often more comprehensive, covering a broader range of information that patients may need. This finding aligns with previous research, which suggests that the chatbot holds significant potential for providing basic patient education. The patient review panel’s results also indicate that the chatbot’s performance in patient education closely mirrors that of physicians, suggesting that the chatbot can offer patient-approved informational support in telemedicine, particularly in alleviating the burden of nonurgent consultations in the context of physician shortages.

The medical review panel believed the chatbot demonstrated more empathy, which may be related to the completeness of its responses. The chatbot’s responses not only included disease-related information but also provided additional suggestions, such as psychological comfort, lifestyle recommendations, and reminders for regular check-ups. In contrast, the physicians’ responses typically focused more on disease-related content. It is also possible that these additional details were discussed by the physicians with the patients in person but were not mentioned in the asynchronous text-based messaging records. On the other hand, the patient review panel expressed the opposite view, considering the chatbot to be lacking empathy. Although the responses seemed comprehensive, patients may have struggled to identify key information, leading to confusion. This highlights the differences in how physicians and patients interpret medical information and suggests that the chatbot’s lengthy responses may create a false sense of empathy, ultimately leaving patients seeking medical support feeling confused. Therefore, the relationship between response completeness and empathy may not be a direct causal one and could differ depending on the perspectives of physicians and patients.

Despite the chatbot’s strong performance in patient education, its capabilities in handling complex medical decision-making remain inadequate. The findings from the medical review panel indicate that, while the chatbot is comparable to physicians in terms of safety, its accuracy is notably poorer, particularly when required to integrate personal medical histories, physical examination results, and other complex factors. One major type of error observed was the misinterpretation of symptoms. For instance, when a patient with a history of breast cancer complained of persistent back pain, the chatbot incorrectly attributed the pain to a musculoskeletal issue. A physician, however, would have considered metastasis, given the patient’s cancer history. This misinterpretation could delay necessary diagnostics, such as imaging or biopsy, potentially worsening the patient’s condition. Another issue was hallucination. For example, the chatbot recommended antidepressants for a patient with late-stage cancer experiencing weight loss, a symptom more likely due to cancer progression than depression. This could have delayed proper treatment, affecting the patient’s health. Feedback from the patient review panel was even more explicit, with patients rating the chatbot as significantly inferior to physicians in terms of the completeness and trustworthiness of its decision-making. This limitation reflects the current technical bottleneck in chatbot performance for complex medical scenarios, particularly in diagnostic or treatment planning tasks, where decision accuracy remains low [29,30]. This starkly contrasts with the chatbot’s strong performance in consultations and emphasizes that, for now, chatbots are better suited as supplementary tools rather than independent decision makers in telemedicine [31].

In addition, it is worth noting that the responses from physicians in this study came from 17 oncologists across different regions of China, with clinical experience ranging from 1.5 to 16 years. The incorrect responses were primarily provided by younger physicians with less experience. As expected, more experienced physicians tend to perform better than novice ones. The average clinical experience of the oncologists participating in this study was 5.2 (SD 4.1) years. If future studies compare the chatbot’s responses with those of more experienced physicians, such as oncologists with at least 10 years of clinical experience, the difference between the two may be more pronounced.

### Interpretation and Mechanisms

This study collected 4257 asynchronous text-based messaging records between physicians and both new and existing patients. Patient demographic information and disease-related information (such as current symptoms, medical examination results, and treatment processes, among others) are crucial for disease diagnosis and treatment. During the first communication with new patients, even if patients fail to provide this information proactively, physicians will actively guide them and record it completely in the medical records. As mentioned in the *Methods* section, the asynchronous text-based messaging records of new patients typically include such personal information in detail. This, to some extent, alleviates the asymmetry in obtaining patient information between oncologists and the chatbot when interacting with new patients.

However, existing patients usually assume that physicians are already well aware of their medical records, and this is indeed the case. Therefore, during telemedicine, existing patients often neglect to provide detailed demographic and disease-related information. Furthermore, because physicians are familiar with the medical records of existing patients, they sometimes overlook the need for patients to supplement personal information. This leads to a situation where when dealing with existing patients, physicians have more information than the chatbot, especially regarding the personal treatment history of existing patients. Even if the chatbot can access complete clinical records, the rich information obtained by physicians through personal interaction still cannot be fully captured by clinical records. Thus, this information asymmetry is difficult to avoid.

Information asymmetry may be one of the factors contributing to the increased likelihood of errors in chatbot responses. For example, in asynchronous text-based messaging exchanges with existing patients, individuals may omit their oncology treatment history since physicians are already familiar with their medical background. When the patient discusses symptoms such as poor appetite or nausea and requests treatment plans, the physician considers whether these symptoms are related to the tumor itself or to chemotherapy or radiation therapy, while the chatbot may only offer advice based on these symptoms, such as suggesting improvements to dietary habits. This clearly demonstrates how the incompleteness of patient information in the chatbot’s dataset can lead to less accurate responses in complex medical decision-making. In addition, all the information acquired by the chatbot comes from the exchange between the patient and the physician. It cannot interact directly with the patient nor actively guide the patient to provide the required information, which may also contribute to the poor performance of the chatbot. Unlike patient education, medical decision-making requires consideration of a broader range of factors, especially personal information, which is often not provided to the chatbot. In view of this, in future interactions with the chatbot, providing as much disease-related information as possible may, to some extent, improve the poor performance of the chatbot caused by information asymmetry.

### Future Directions

While chatbots currently underperform in medical decision-making, further technical optimizations—especially task-specific and scenario-specific fine-tuning—may enhance their decision-making capabilities [32]. Fine-tuning involves additional training of models on datasets containing specific tasks or correct answers [33]. Existing research suggests that broader fine-tuning can significantly improve chatbot accuracy and reduce the occurrence of hallucinations [34]. Future research should continue to explore how targeted fine-tuning can enhance chatbot performance in medical decision-making scenarios.

The deployment of chatbots in telemedicine raises critical concerns regarding privacy, security, and ethics [35]. Since chatbots rely on large datasets for training, it is essential to ensure that patient privacy is protected to prevent the disclosure of personally identifiable information during interactions. In addition, given that patients may find it difficult to assess the accuracy of chatbot responses, physicians have a responsibility to review these responses to ensure their safety and accuracy. This implies that even as chatbot performance improves, they must remain subject to physician oversight and guidance to mitigate potential medical risks [36,37].

Future research should further explore how to optimize chatbot question-and-answer systems while safeguarding patient privacy and data security [38,39]. For example, researchers could focus on designing chatbots that can naturally guide the conversation and ensure the collection of critical medical information through intuitive and patient-friendly interactions. In addition, research should focus on improving the chatbot's response mechanisms to maintain consistency and accuracy across different question formats and sequences, thereby enhancing its clinical applicability.

### Strengths and Limitations

This study has several limitations. First, the focus on patients with cancer, whose disease management is highly specialized, may limit the generalizability of our findings to other medical conditions. Future research should expand the scope to explore chatbot performance in other clinical settings. Second, although our study used simulated, asynchronous, text-based messaging scenarios designed to closely replicate real-world clinical communication, these simulations remain distinct from actual clinical practice, which may introduce bias when applying the findings to real-world settings. Future studies could address this by conducting large-scale trials in real clinical environments to validate the practical application of chatbots. Third, a fundamental limitation of our methodology is that the chatbot did not interact with patients in real-time clinical conversations. Since the chatbot was unable to request clarification or obtain additional information from patients, its responses might not accurately reflect the dynamic nature of actual clinical interactions. Future research could address this limitation by designing studies with real-time patient-chatbot interactions.

### Conclusion

In conclusion, this study evaluated the performance of chatbot in different clinical scenarios within telemedicine. The results indicate that while the chatbot excels in providing patient education, they lack the capacity to replace physicians in making medical decisions. Furthermore, their integration into clinical practice requires stringent medical supervision, with careful consideration of privacy and ethical concerns.

## Appendix

Multimedia Appendix 1 STROBE checklist.

Multimedia Appendix 2 METRICS checklist.

## Abbreviations

LLM: large language model
METRICS: Model, Evaluation, Timing, Range/Randomization, Individual factors, Count, and Specificity
STROBE: Strengthening the Reporting of Observational Studies in Epidemiology
WHO: World Health Organization

## Data Availability

The datasets generated or analyzed during this study are available from the corresponding author on reasonable request.
