# Supplementary material for: Application of AI Chatbot in Responding to Asynchronous Text-Based Messages From Patients With Cancer: Comparative Study
Source: J Med Internet Res. 2025 May 21;27:e67462. doi: 10.2196/67462 (PMC12138309; doi:10.2196/67462)
Supplement: Multimedia Appendix 2 [file jmir_v27i1e67462_app2.doc]

METRICS Statement—checklist

|  | Item No | Recommendation |
| --- | --- | --- |
| **Model** | 1 | What is the model of the generative AIa tool used for generating content, and what are the exact settings for each tool? |
| **Evaluation** | 2 | What is the exact approach used to evaluate the content generated by the generative AI-based model and is it an objective or subjective evaluation? |
| **Timing** | 3 | (*a*) *Timing*—When is the generative AI model tested exactly and what are the duration and timing of testing? |
|  |  | (*b*) *Transparency*—How transparent are the data sources used to generate queries for the generative AI-based model? |
| **Range** | 4 | (*a*) *Range*—What is the range of topics tested and are they intersubject or intrasubject with variability in different subjects? |
|  |  | (*b*) *Randomization*—Was the process of selecting the topics to be tested on the generative AI-based model randomized? |
| **Individual** | 5 | Is there any individual subjective involvement in generative AI content evaluation? If so, did the authors describe the details in full? |
| **Count** | 6 | What is the count of queries executed (sample size)? |
| **Specificity of the prompt or language** | 7 | How specific are the exact prompts used? Were those exact prompts provided fully? Did the authors consider the feedback and learning loops? How specific are the language and cultural issues considered in the generative AI model? |

**Reference:** Sallam M, Barakat M, Sallam M

A Preliminary Checklist (METRICS) to Standardize the Design and Reporting of Studies on Generative Artificial Intelligence–Based Models in Health Care Education and Practice: Development Study Involving a Literature Review

Interact J Med Res 2024;13:e54704

doi: 10.2196/54704

PMID: 38276872

PMCID: 10905357
